# Cervicovaginal Mycobiome Restructuring by HPV and Bacterial Community State Types in a Kazakhstani Shotgun Metagenomic Cohort: *Lactobacillus iners* as a *Candida*-Permissive Niche Associated with α-9 HPV in Cytologically Normal Women

**DOI:** 10.3390/ijms27115052

**Published:** 2026-06-03

**Authors:** Samat Kozhakhmetov, Almagul Kushugulova, Elizaveta Vinogradova, Aidana Rakhmankulova, Milan Terzic, Gauri Bapayeva, Gulzhanat Aimagambetova, Nazira Kamzayeva, Yevgeniy Kim, Berik Primbetov, Balkenzhe Imankulova, Kuralay Kongrtay, Nazira Kadroldinova, Makhabbat Galym, Sanimkul Makhambetova, Kadisha Nurgaliyeva, Zhanar Abdiyeva, Zhanar Zhumakanova, Dana Baktybayeva, Balnur Smagulova, Talshyn Ukybassova

**Affiliations:** 1 Laboratory of Microbiome, Center for Life Sciences, National Laboratory Astana, Nazarbayev University, 53 Kabanbay Batyr Ave., Block S1, Astana Z05H0P9, Kazakhstanst.paulmississippi@gmail.com (E.V.); aidana.rakhmankulova@nu.edu.kz (A.R.); 2 Department of Normal Physiology, Sechenov First Moscow State Medical University, 119991 Moscow, Russia; 3 Clinical Academic Department of Women’s Health, Corporate Fund “University Medical Center”, Turan 32, Astana 010000, Kazakhstanevg.kim94@gmail.com (Y.K.); imanbalken@mail.ru (B.I.); nazira.kadroldinova@nu.edu.kz (N.K.); dana_baktybaeva@mail.ru (D.B.); 202514637@amu.kz (B.S.);; 4 Interdisciplinary Sports Research, Center for Genetics and Life Sciences, Sirius University of Science and Technology, 1 Olympic Ave., Sirius Federal Territory, 354340 Sochi, Russia; 5 Department of Biomedical Sciences, School of Medicine, Nazarbayev University Kerei-Zhanibek Khandar Street, 5, Astana Z05K4F4, Kazakhstan; 6 Department of Surgery, School of Medicine, Nazarbayev University, Kerei-Zhanibek Khandar Street, 5/1, Astana Z05K4F4, Kazakhstan

**Keywords:** cervicovaginal microbiome, cervicovaginal mycobiome, human papillomavirus (HPV), *Lactobacillus iners*, *Candida*, shotgun metagenomics, bacterial–fungal cross-kingdom interactions, Central Asia

## Abstract

Cervicovaginal dysbiosis is an established co-factor of high-risk human papillomavirus (HPV) persistence and cervical neoplastic development, yet most studies address the bacterial compartment in isolation, leaving fungal communities and bacterial–fungal cross-kingdom interactions underexplored, particularly in Central Asian populations. We performed shotgun metagenomic sequencing (mNGS) of cervicovaginal samples from 311 Kazakhstani women undergoing routine cervical screening. HPV status was determined using combined PCR and mNGS methods, and cervical screening was completed using liquid-based cytology (NILM, ASC-US, LSIL, ASC-H). Bacterial, viral, and fungal taxa were profiled from a single shotgun dataset with Kraken2 pipeline. Bacterial community state types (CSTs) were determined based on dominant bacterial species, functional gene content was annotated against KEGG using eggNOG, and covariate-adjusted associations were estimated using MaAsLin3. Mycobiome β-diversity differed significantly by HPV status (*p* = 0.003). In particular, *Candida* positivity was significantly associated with HPV presence and with high-risk α-9 HPV in cytologically normal (NILM) samples (OR = 3.6, [1.6–9.6], *p* ≤ 0.001). Covariate-adjusted analysis was consistent with this positive association (q < 0.05). Concurrently, among CSTs, *Lactobacillus iners*-dominated CST III and dysbiotic *Gardnerella vaginalis*-dominated CST IV showed a 3-fold higher *Candida albicans* prevalence (*p* < 0.01). Further analysis demonstrated that, functionally, both of these CSTs had depleted capacity for lactate metabolism (ko00620, *p* < 0.0001) and, in particular, for the genetic capacity for pyruvate-dependent H_2_O_2_ generation (half that of the *L. crispatus*-dominated CST I). These findings support *L. iners* as a metabolically permissive rather than protective *Lactobacillus* and suggest cross-kingdom functional signatures as candidate biomarkers for HPV acquisition and persistence in Central Asia, a region previously absent from the cervicovaginal microbiome literature.

## 1. Introduction

Cervical cancer remains the fourth most common malignancy in women globally, with an estimated 662,000 new cases and 349,000 deaths in 2022, with the highest age-standardized rates concentrated in low- and middle-income settings [1]. Persistent infection with high-risk human papillomavirus (HR-HPV) is the necessary causal agent, and within the alpha genus of the *Papillomaviridae,* the α-9 phylogenetic species—HPV-16 and its relatives HPV-31, -33, -35, -52, -58—carries the highest oncogenic potential [2,3]. Only a small fraction of HPV infections progress to neoplasia, and cervicovaginal bacterial dysbiosis has emerged as a major co-factor modulating viral persistence, local immune activity, and epithelial barrier integrity [4,5,6]. Over the past decade, bacterial community state types (CSTs I-V) defined by *Lactobacillus* species dominance [7,8,9] have become the standard analytical frame: *L. crispatus*-dominated CST I is reproducibly associated with viral clearance and epithelial protection, whereas *L. iners*-dominated CST III and *Lactobacillus*-deficient, *Gardnerella*-enriched CST IV consistently track with HPV persistence and cytological abnormality [10,11,12].

Evidence is emerging, however, of important gaps in this bacteria-centric framework. First, the fungal compartment of the cervicovaginal niche—the mycobiome—remains underexplored relative to its bacterial counterpart, despite accumulating evidence that *Candida* species may themselves modify local immunity and epithelial integrity [13]. Cervicovaginal fungi have historically been studied almost exclusively in the context of vulvovaginal candidiasis, with *C. albicans* dominating clinical attention, whereas culture-independent surveys using ITS amplicon sequencing and, more recently, shotgun metagenomics and metaproteomics have revealed a considerably more diverse community [14,15], including non-albicans *Candida* species (*C. glabrata*, *C. parapsilosis*, *C. dubliniensis*), basidiomycete yeasts (*Malassezia*, *Cryptococcus*, *Rhodotorula*), and taxa whose cervicovaginal residency remains uncertain. Second, even within the bacterial compartment, taxonomic identity at the genus or species level often fails to capture the functional heterogeneity that actually determines ecological impact. *L. iners* is a paradigmatic case; it belongs to the *Lactobacillus* genus but has a genome markedly smaller than *L. crispatus* and lacks several pathways central to the classical protective phenotype, including robust production of D-lactate and hydrogen peroxide [16,17].

Emerging evidence further suggests that linking bacterial and fungal compartments mechanistically requires moving beyond taxonomic co-occurrence to the functional repertoire of each community [18]. The protective bacterial phenotype classically attributed to *Lactobacillus* rests on at least three mechanisms with direct antifungal and antiviral relevance: lactic acid production, particularly D-lactate, which maintains vaginal pH below 4.5 and inhibits matrix metalloproteinases involved in epithelial remodeling [19,20]; hydrogen peroxide production via pyruvate oxidase, which exerts direct antimicrobial activity and contributes to colonization resistance against both bacterial and fungal competitors [21]; and competitive exclusion at the epithelial surface through adhesion and biofilm formation [22]. *L. crispatus* possesses all three capabilities at high functional abundance, whereas *L. iners* produces predominantly L-lactate with limited D-lactate and lacks efficient hydrogen peroxide production [16]—a functional asymmetry increasingly invoked to explain the well-documented clinical observation that *L. iners*-dominated communities provide markedly less protection against HPV persistence and cervical abnormality than *L. crispatus*-dominated ones [11,23]. Acidic pH and reactive oxygen species are both established inhibitors of fungal growth in the vaginal niche, and their depletion is expected to create a metabolically permissive environment for opportunistic fungi, most notably *Candida* species, independently of the broader dysbiotic signature captured by CST IV.

The epithelial consequences of *Candida* colonization provide a biological bridge to HPV acquisition. *C. albicans* produces candidalysin, a cytolytic peptide cleaved from the ECE1 gene product that permeabilizes epithelial membranes and activates pro-inflammatory signaling [24,25]. A mucosal surface repeatedly exposed to *C. albicans* in this active form may present a lower effective barrier to HPV acquisition and persistence, particularly for α-9 high-risk types whose transmission requires microtrauma and access to basal epithelial cells [26]. A direct test of these predictions requires paired taxonomic and functional profiling of the bacterial community alongside systematic characterization of the fungal compartment, an approach that remains rare in the cervicovaginal microbiome literature.

The dominant fungal genera reported in the cervicovaginal niche appear to vary systematically across populations. In a Caribbean Hispanic cohort, *Sporidiobolaceae*, *Malassezia*, and *Candida* each emerged as dominant taxa in distinct subsets of women, with fungal diversity elevated in HR-HPV-positive samples. In a South African cohort studied by metaproteomics, *Candida* dominated the mycobiome and *Malassezia* proteins were enriched in bacterial vaginosis [15,27]. In a Costa Rican longitudinal cohort, fungal diversity was associated with protection against CIN2+ progression [27], and a large Chinese cohort reported a dual effect of *Candida* on HPV, protective against acquisition cross-sectionally but promoting persistence longitudinally [28]. Whether these patterns reflect true population-level biology, differences in methodology and reference databases, or environmental exposures remains an open question, and, to our knowledge, no cervicovaginal mycobiome study has been reported from Central Asia.

The overwhelming majority of cervicovaginal microbiome studies to date have sampled women of East Asian, European, African, or Latin American ancestry. Central Asian populations, including Kazakhstan—a country with a mixed Turkic, Russian, and Central Asian demographic profile, a cervical cancer age-standardized incidence rate of approximately 19 per 100,000 women (around three-fold higher than rates reported for Western Europe, 7.3, and North America, 6.6 [1], a substantial HPV burden with published prevalence estimates of 25–55% in different population- and clinic-based cohorts [29], and an opportunistic rather than fully organized screening program—remain essentially unrepresented in the published cervicovaginal microbiome literature [30]. This combination makes Kazakhstan a strategically important site for filling the geographic gap in cervicovaginal microbiome research.

Considering these three lines of evidence—gaps in characterization of the cervical mycobiome, the insufficiency of isolated CST-based functional bacterial profiling, and the near-complete absence of Central Asian data—the aim of the present study was to characterize, for the first time in a Central Asian population, the cervicovaginal mycobiome and its bacterial–fungal cross-kingdom interactions in relation to HPV status and cervical cytology using single-assay shotgun metagenomics, and to integrate taxonomy with KEGG-based functional profiling. Specifically, we sought (i) to characterize cervical mycobiome of Kazakhstani women and to test whether the cervicovaginal mycobiome is systematically restructured by HPV status in a Kazakhstani cohort and to identify class- and species-level fungal markers of HPV positivity; (ii) to quantify the association of *Candida* colonization with HPV in general and with high-risk α-9 HPV in particular, stratified by cervical cytology, to assess the timing of the cross-kingdom signal relative to cervical abnormality; and (iii) to determine whether *L. iners*-dominated CST III carries a distinct functional signature at the level of pyruvate metabolism, H_2_O_2_ biosynthesis (K00158), and genetic potential for D-lactate production (K03778) that distinguishes it from *L. crispatus* protection and brings it closer to *Gardnerella*-associated dysbiosis, and whether that signature coincides with elevated fungal prevalence.

## 2. Results

### 2.1. Cohort Overview and HPV Stratification

The final analytical sample comprised 311 reproductive-age women (median age 34.51 (IQR: 30.46; 39.2) years; BMI 22.84 (IQR: 20.43; 25.72); 90.8% ethnic Kazakh). Distribution by four Bethesda cytological categories was the following: NILM (*n* = 145), ASC-US (*n* = 75), LSIL (*n* = 81), and ASC-H (*n* = 10). HPV positivity showed a pronounced association with cytological severity (OR = 4.4 [2.7–7.4], *p* ≤ 0.0001), confirming the biological validity of the stratification (Table 1).

HPV-negative cases had higher parity (2.0 (IQR: 1.0; 3.0) vs. 1.0 (IQR: 0.0; 2.0), *p* ≤ 0.001) and were significantly more likely to be married (74.7% vs. 55.5%, *p* ≤ 0.001).

### 2.2. The Cervicovaginal Mycobiome Is Restructured by HPV Status (Figure 1)

**Figure 1:**
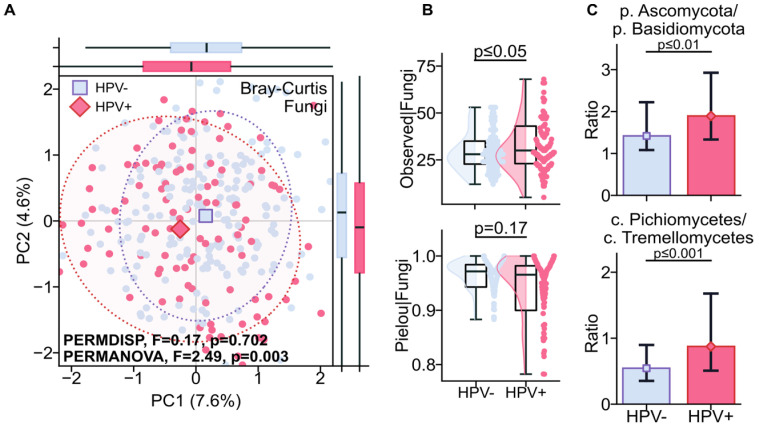
Fungal mycobiome composition stratified by HPV status (*n* = 311 for CST-inclusive analyses; *n* = 311 for cytology-stratified subgroups). (**A**) Principal coordinates analysis on Bray–Curtis distances shows significant separation of HPV- (light blue) and HPV+ (pink) fungal communities; PERMANOVA F = 2.49, *p* = 0.003; PERMDISP F = 0.17, *p* = 0.702 (dispersion preserved, confirming a true centroid shift). (**B**) Fungal alpha diversity: observed species richness is significantly elevated in HPV+ (*p* ≤ 0.05), whereas Pielou’s evenness does not differ (*p* = 0.17). (**C**) Phylum- and class-level ratios: *Ascomycota/Basidiomycota* (*p* ≤ 0.01) and *Pichiomycetes*/*Tremellomycetes* (*p* ≤ 0.001, the most sensitive fungal marker of HPV status).

Shotgun metagenomic profiling recovered a compact fungal signal across the cohort, despite the low fungal biomass characteristic of the cervicovaginal niche. Fungal community composition differed significantly between HPV-negative and HPV-positive women (Figure 1A; PERMANOVA F = 2.49, *p* = 0.003 on Bray–Curtis distances of Hellinger-transformed abundances). Dispersion homogeneity was preserved (PERMDISP F = 0.17, *p* = 0.702), indicating that the observed separation reflects a centroid shift rather than an artefact of heterogeneity.

Alpha diversity showed a selective expansion of the fungal repertoire in HPV-positive women. Observed species richness was significantly elevated at HPV+ (*p* ≤ 0.05), whereas Pielou’s evenness did not differ between groups (*p* = 0.17; Figure 1B). This suggests that HPV infection, therefore, broadens the fungal taxonomic spectrum, a signature compatible with opportunistic expansion of low-abundance fungi rather than wholesale community restructuring.

At higher taxonomic levels, two ratios captured consistent HPV-associated shifts. The *Ascomycota*/*Basidiomycota* ratio was significantly higher in HPV-positive women (*p* ≤ 0.01). The class-level *Pichiomycetes*/*Tremellomycetes* ratio, which contrasts ascomycete yeasts with basidiomycete yeasts, showed an even stronger difference and was the most sensitive fungal marker of HPV status in the entire analysis (*p* ≤ 0.001; Figure 1C). We note that in current NCBI taxonomy, the genus *Candida* is nested within the class Pichiomycetes (order *Serinales*, subphylum *Saccharomycotina*, phylum *Ascomycota*). The *Pichiomycetes*/*Tremellomycetes* ratio reported here, therefore, partially overlaps with the species-level *Candida* enrichment described in Section 2.3 and is presented as a class-level summary statistic of the *Ascomycota*/*Basidiomycota* yeast balance rather than as a *Candida*-independent observation. This class-level metric collapses compositional shifts onto a single interpretable axis and may offer a candidate translational biomarker, pending validation in independent cohorts.

### 2.3. Candida Colonization Is Associated with HPV, Particularly with High-Risk α-9 HPV, and the Effect Is Strongest in Cytologically Normal Samples (Figure 2)

**Figure 2:**
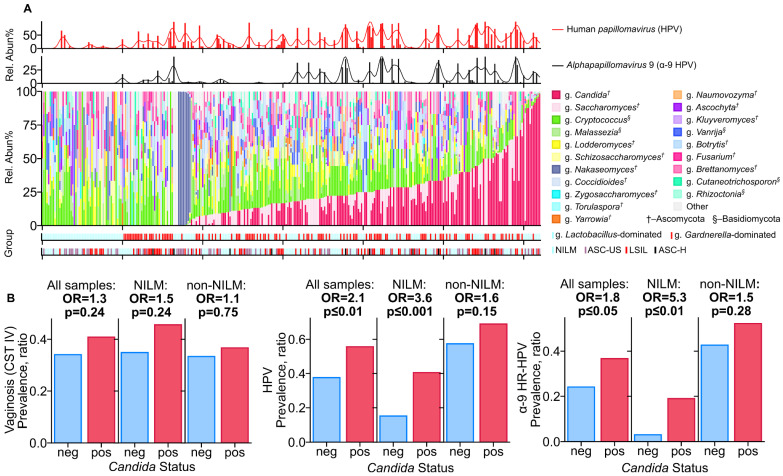
Taxonomic landscape of the cervicovaginal mycobiome and association of *Candida* colonization with HPV and high-risk α-9 HPV (*n* = 311). (**A**) Stacked genus-level relative abundance bar plot (human-associated fungi only), with per-sample HPV (red) and α-9 HPV (black) relative abundance tracks shown above, and bacterial group (*Lactobacillus*-dominated vs. *Gardnerella*-dominated) and Bethesda cytological category (NILM, ASC-US, LSIL, ASC-H) legends below. (**B**) Bar plot of HPV prevalence by *Candida* status (negative vs. positive) in all samples, stratified by cytological status (NILM—no cervical changes vs. non-NILM—samples with abnormal cytology, including ASC-US, LSIL, and ASC-H). Odds ratios and *p*-values are shown for all samples, NILM, and non-NILM subsets. Fisher’s exact test, unadjusted for covariates, with no correction for multiple comparisons.

To identify which fungal taxa are associated with the HPV-associated restructuring described above, we examined the genus-level mycobiome landscape after restriction to human-associated fungi (Methods). Across the cohort, *Candida* was the dominant genus in a substantial subset of samples (Figure 2A, stacked community bars), with *Saccharomyces*, *Cryptococcus*, and *Lodderomyces* forming secondary dominance patterns. Our cohort showed a prominent *Cryptococcus* signal as a basidiomycete component, in contrast to Caribbean Hispanic cohorts where *Malassezia* has been reported as the dominant basidiomycete. The per-sample community structure aligned with the quantitative linear tracks positioned above the stacked bars: samples with elevated *Candida* relative abundance were enriched for both total HPV (red track) and *Alphapapillomavirus* 9 (black track).

Positioning these Kazakhstani observations against the published cervicovaginal mycobiome literature (Table 2) shows that *Candida* (*Ascomycota*, *Saccharomycetes*) is a reproducible component of the cervicovaginal mycobiome across every population examined by sequencing or metaproteomics to date, while the identity of the dominant Basidiomycota varies substantially by region: *Sporidiobolaceae* and *Malassezia* in Caribbean Hispanic cohorts, *Malassezia* in South African cohorts, and *Cryptococcus* (class *Tremellomycetes*) in the present Kazakhstani cohort. To our knowledge, *Tremellomycetes* prominence has not previously been reported for the cervicovaginal niche.

To formalize this observation, we calculated odds ratios for three clinically relevant outcomes as a function of *Candida* status (positive vs. negative at the genus level), both across the full cohort and stratified by cytological normality (NILM vs. non-NILM; Figure 2B). Three patterns emerged.

First, the association between *Candida* and cytologically defined vaginosis (CST IV) was weak and non-significant in the cohort as a whole (OR = 1.3 [0.8–2.1], *p* = 0.24), within NILM (OR = 1.5 [0.8–3.2], *p* = 0.24) and in non-NILM (OR = 1.1 [0.6–2.2], *p* = 0.75). *Candida* colonization was, therefore, not strongly predicted by bacterial dysbiosis alone.

Second, *Candida* colonization was significantly associated with overall HPV positivity (OR = 2.1 [1.3–3.4], *p* ≤ 0.01), and the effect was considerably stronger in the NILM stratum (OR = 3.6 [1.6–9.6], *p* ≤ 0.001) than among non-NILM women (OR = 1.6 [0.9–3.4], *p* = 0.15). Within NILM, HPV prevalence rose from 15% (*n* = 10) in *Candida*-negative samples to 40% (*n* = 32) in *Candida*-positive samples, a 2.5-fold absolute increase.

Third, the association with *Alphapapillomavirus* 9, the phylogenetic cluster including HPV-16 and closely related high-risk types, was substantially stronger: OR = 1.8 [1.1–3.0] across the cohort (*p* ≤ 0.01), OR = 5.3 [1.6–69.5] within NILM (*p* ≤ 0.01), and OR = 1.5 [0.7–2.8] in non-NILM (*p* = 0.28). In cytologically normal women, α-9 HPV prevalence rose from 3.0% (*n* = 2) in *Candida*-negative samples to 19% (*n* = 15) in *Candida*-positive samples, yielding a nearly 6-fold increase despite the small absolute numbers.

This cross-stratum pattern admits two interpretations. First, the *Candida*-HPV signal reaches its maximum in cytologically normal women, that is, before cervical abnormality has appeared. Second, baseline HPV prevalence in non-NILM samples is already high (107/166 = 64.5%) and rises only modestly in *Candida*-positive non-NILM women (70%, *n* = 63), an approximately six-percentage-point increase that does not reach significance (OR = 1.6 [0.9–3.4]). The compressed dynamic range above this elevated baseline partially obscures any *Candida*-associated risk in the non-NILM stratum, in contrast to the NILM stratum where prevalence rises 2.5-fold and the OR reaches 3.6. This placement is consistent with our earlier finding that fungal dysbiosis tracks HPV status but not cytological category, and together the two observations are compatible with co-occurrence of elevated *Candida* prevalence and HPV positivity in cytologically normal women, prior to the appearance of cervical lesions. The cross-sectional design does not permit ordering of the *Candida*–HPV sequence, and this question requires longitudinal follow-up.

### 2.4. Bacterial Community State Types Structure the Mycobiome via Distinct Metabolic Repertoires (Figure 3)

The analyses above establish two findings: an HPV-associated restructuring of the cervicovaginal mycobiome (Section 2.2) and a *Candida*-HPV association that is strongest in cytologically normal samples and is not accounted for by vaginosis (*Gardnerella*-dominated CST IV) alone (Section 2.3). If non-optimal bacterial composition at the level of CST is not sufficient to explain the *Candida* signal, the relevant bacterial feature must lie below the level of taxonomic classification. To examine this, we complemented CST-based stratification with KEGG functional annotation of the bacterial metagenome and tested fungal prevalence as a function of CST-specific metabolic capacity.

**Figure 3:**
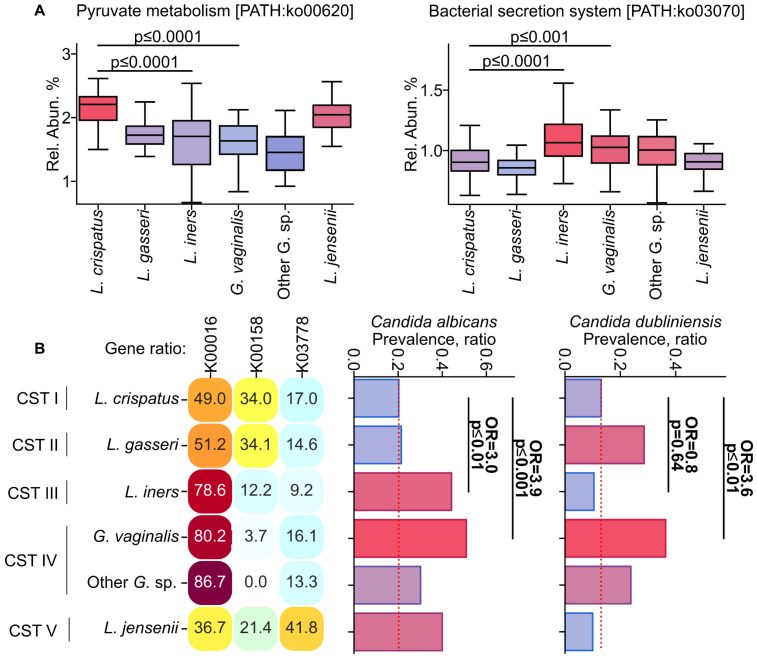
Bacterial community state types structure the cervicovaginal mycobiome through distinct metabolic repertoires. (**A**) KEGG pathway-level relative abundance across bacterial CSTs for pyruvate metabolism (ko00620, **left**) and bacterial secretion system (ko03070, **right**). *L. crispatus* and *L. jensenii* communities show elevated pyruvate metabolism, whereas *L. iners* and *G. vaginalis* communities show elevated secretion system abundance. Pairwise comparisons against *L. crispatus*: *p*-values annotated. Box colours encode the CST-defining taxon and are kept consistent throughout the figure. (**B**) **Left**: median gene ratio in each CST for K00016 (L-lactate dehydrogenase, which may operate in lactate-producing or lactate-utilizing direction depending on organism), K00158 (pyruvate oxidase, H_2_O_2_-generating), and K03778 (D-lactate dehydrogenase). **Right**: *C. albicans* and *C. dubliniensis* presence. Heatmap colour intensity is proportional to the median gene ratio per KO; the red dashed line marks the cohort-wide prevalence of each Candida species as a reference for per-CST enrichment. Fisher’s exact test, unadjusted for covariates, with no correction for multiple comparisons.

Pathway-level analysis demonstrated significant redistribution in the relative abundance of key KEGG pathways with CST-specific patterns (Figure 3A). The pathway of lactate metabolism is ko00620 (pyruvate metabolism). The pyruvate metabolism pathway gene abundance, which includes both L- and D-lactate dehydrogenase branches alongside other fermentative reactions, was enriched in *L. crispatus*-dominated communities (median 2.21% (IQR: 1.96; 2.33)) and *L. jensenii*-dominated communities (2.04% (IQR: 1.85; 2.20)), relative to *L. iners* (1.71% (IQR: 1.26; 1.95)), *G. vaginalis* (1.63% (IQR: 1.43; 1.87)), and other *Gardnerella* species (1.45% (IQR: 1.17; 1.70)). Contrasts of *L. crispatus* with *L. iners* and with *G. vaginalis* were highly significant (both *p* ≤ 0.0001). The bacterial secretion system pathway (ko03070), which can be broadly associated with virulence capacity, showed the opposite pattern: *L. iners*-dominated communities had significantly elevated abundance of secretion system genes (1.07% (IQR: 0.95; 1.22)) compared with *L. crispatus* (0.9% (IQR: 0.83; 1.00); *p* ≤ 0.0001) and *L. gasseri* (0.86% (IQR: 0.8; 0.92); *p* ≤ 0.0001), and *G. vaginalis* communities showed a similar elevation (1.03% (IQR: 0.9; 1.12); *p* ≤ 0.01). This bimodal functional profile, combining reduced pyruvate metabolism pathway abundance with elevated secretion systems, is compatible with a pro-inflammatory, barrier-permeabilizing bacterial phenotype in *L. iners* and *G. vaginalis* communities and is not captured by species-level identity alone.

Gene-level analysis further resolved the functional divergence between *Lactobacillus* species (Figure 3B, heatmap). We examined three KEGG orthology genes directly relevant to bacterial protection of the cervicovaginal niche. The ratio of K00016 (L-lactate dehydrogenase)/K03778 (D-lactate dehydrogenase)/K00158 (pyruvate oxidase, H_2_O_2_-generating) genes in the ko00620 pathway was relatively even in CST I (*L. crispatus*)-dominated samples at 49:34:17, and in CST II, this proportion was approximately similar 51:34:14. In CST III (*L. iners*), however, the proportion showed a significant skew of 78:12:9. This skew is consistent with the documented gene-level impairment of D-lactate (K03778) and H_2_O_2_ (K00158) biosynthesis in *L. iners* and indicates that CST III communities carry reduced gene-level capacity for H_2_O_2_ generation—a function that has been proposed to contribute to colonization resistance in *L. crispatus* and *L. gasseri*. Since D-lactate has been shown to inhibit matrix metalloproteinase activity in the cervicovaginal epithelium, its depletion in *L. iners*-dominated communities may contribute to reduced epithelial protection, although direct measurement of D-lactate concentrations and MMP activity was beyond the scope of this study. In samples dominated by *G. vaginalis* and related *Gardnerella* species, the community showed an even greater skew in the lactate metabolism gene pool, with less than 5% falling to H_2_O_2_ (K00158).

These findings indicate not only that *L. iners* and *Gardnerella*-dominated communities lack the potential for lactate metabolism (ko00620) but they also demonstrate lower potential, specifically for D-lactate and H_2_O_2_ production. Notably, the detected potential for lactate metabolism with gene skew in *Gardnerella*-dominated communities can be explained by preferential co-occurrence with permissive *Lactobacillus* species (such as *L. iners*).

Taken together, CST III-dominated samples demonstrated a distinct functional signature: retained L-lactate production capacity, reduced H_2_O_2_ and D-lactate capacity, and an increased proportion of secretion system capacity genes associated with virulence. This configuration differs from the protective profile of CST I, in which all three protective functions are retained at high levels, and is more similar to dysbiotic CST IV. This profile is not necessarily that of a deficient *Lactobacillus*; rather, it is the profile of a functionally distinct, metabolically restructured *Lactobacillus* lineage.

Fungal prevalence mapped onto these functional CSTs in an ecologically coherent pattern (Figure 3B, right panels). Relative to *L. crispatus*-dominated CST I, *C. albicans* prevalence was elevated in non-*L. crispatus* CSTs, with odds ratios of 3.0 [1.4–7.0] for CST III (*L. iners*) (*p* = 0.01) and 3.9 [1.7–9.7] for CST IV-B (*Gardnerella*) (*p* ≤ 0.01), representing a 3- to 3.9-fold increase in *C. albicans* prevalence. The *C. dubliniensis*-specific prevalence signal, however, was different: OR = 0.8 [0.3–2.4] for CST III (*p* ≤ 0.64) and OR = 3.6 [1.5–10.5] for CST IV (*p* ≤ 0.01). The magnitude of the *C. albicans* signal in CST III, nearly equal to that in CST IV-B, matches the gene-level finding: *L. iners* communities, despite retaining the *Lactobacillus* taxonomic label, exhibit fungal permissiveness approaching the classically dysbiotic *Gardnerella* state.

Notably, these co-occurrence data suggest that an *L. iners*-dominated community may still inhibit *C. dubliniensis* but not *C. albicans*, consistent with reports that *C. dubliniensis* has a weaker stress response mechanism. In particular, *C. albicans* is known to possess more robust acid tolerance and oxidative stress responses, enabling it to persist in *L. iners* communities, whereas *C. dubliniensis* may require an even more permissive environment typical of CST IV [34]. However, inhibition ability was not directly assessed in this study.

### 2.5. Differential Association of Specific Fungal Taxa with HPV: MaAsLin3 Analysis (Figure 4)

To extend the ecological picture to individual fungal taxa while adjusting for demographic and clinical covariates, we applied MaAsLin3 using the prevalence model, which tests presence/absence associations independent of abundance levels. The model was adjusted for age, BMI, parity, menstrual cycle phase, cell status, marital status and number of sexual partners. We tested seven fungal taxa spanning two genera: *Candida* (with three species: *C. albicans*, *C. dubliniensis*, and *C. orthopsilosis*) and *Saccharomyces* (with *S. cerevisiae.* and *S. paradoxus*). Associations were examined against four predictors: HPV-positive status, α-9 HR-HPV, *Lactobacillus iners*-dominated CST, and *Gardnerella vaginalis*-dominated CST.

**Figure 4:**
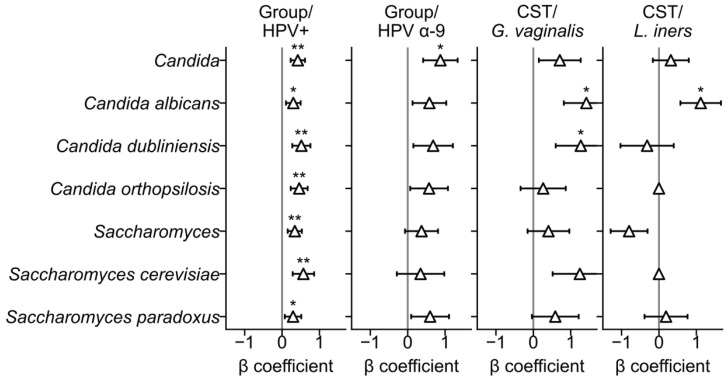
MaAsLin3 prevalence model β-coefficients with standard errors (horizontal whiskers). Taxa tested: *Candida* genus; *C. albicans*; *C. dubliniensis*; *C. orthopsilosis*; *Saccharomyces* genus; *S. cerevisiae*, *S. paradoxus*. Predictors: HPV-positive status, α-9 HPV, *Gardnerella vaginalis*-dominated CST, *Lactobacillus iners*-dominated CST. The vertical line at β = 0 indicates no association. The model was adjusted for age, BMI, parity, menstrual cycle phase, cell status, marital status and number of sexual partners. FDR-adjusted significance: * q < 0.05; ** q < 0.01.

All of the fungal taxa tested were positively and significantly associated with HPV-positive status at q < 0.05. This pattern indicates that the HPV-fungal associations we reported at the community level (Section 2.2) and at the genus level (Section 2.3) persist after adjustment for demographic and clinical covariates and extend down to the species level for the two most commonly reported vaginal yeast genera. However, contrary to pan-HPV, the α-9 HR-HPV presence stratification demonstrated fewer significant associations, mainly associated with *Candida* prevalence in general and not specific species.

At the same time, CST-stratified associations showed species-level divergence that would be missed by genus-level reporting. In *Gardnerella vaginalis*-dominated CST IV, the two *Candida* species associations were significant: *C. albicans* (q < 0.05) and *C. dubliniensis* (q < 0.05). However, neither the *Candida* genus aggregate nor *C. orthopsilosis* reached significance in this CST, indicating that the *G. vaginalis*–fungal association might be species-specific to *C. albicans* and its close phylogenetic relative *C. dubliniensis*, rather than a broader *Candida* phenomenon. In *L. iners*-dominated CST III, only *C. albicans* showed a positive association (q < 0.05), while the association with *C. dubliniensis* was insignificant, supporting the observation in Section 2.4, regarding *C. dubliniensis’s* lower virulence.

These covariate-adjusted associations converge with the community-level (Section 2.2), CST-stratified (Section 2.3), and functional (Section 2.4) findings. Across three analytical angles (community dissimilarity, cross-kingdom co-occurrence, and covariate-adjusted association), *Candida* colonization is repeatedly associated with HPV positivity, in particular with the α-9 HR-HPV clade, and is amplified in non-optimal or dysbiotic CSTs.

### 2.6. Exploratory Analysis of Candidalysin (ECE1) Gene Detection

Given the strong *Candida*-HPV association identified in Section 2.3, we performed a preliminary targeted search of the metagenomic reads for the *Candida albicans ECE1* gene, which encodes the precursor of candidalysin, a cytolytic peptide capable of permeabilizing epithelial membranes. *ECE1* sequences were detected above the minimum read and identity thresholds in eight samples (*n* = 8/311, 2.57%). A broader search including ECE1 together with associated genes (see Methods section for the list), including secreted aspartyl proteinase (SAP) family genes, yielded positive signals in 17 samples (*n* = 17/311, 5.47%).

These numbers are too small to support independent statistical inference in the present cohort. The recovery of ECE1 in 8 of 109 *C. albicans*-positive samples should be interpreted as a sensitivity limit rather than as biological absence: untargeted shotgun sequencing of a single-copy ~1.7 kb gene in a low-biomass niche (median 132 fungal-classified reads per sample) provides a limited statistical opportunity for gene-level detection, and targeted ECE1 enrichment or PCR-based validation will be required to estimate true carriage prevalence. The same targeted search was reference-anchored to the *C. albicans ECE1* sequence; species-specific annotation for *C. dubliniensis* and other related taxa is a separate question that is left for follow-up work. The ability to recover *ECE1* sequences from routine shotgun data without fungal-specific enrichment, nonetheless, demonstrates feasibility and motivates targeted profiling in future work.

## 3. Discussion

This shotgun metagenomic study of cervicovaginal samples from 311 Kazakhstani women is, to our knowledge, the first cervicovaginal mycobiome characterization from Central Asia. This study produced several interconnected findings that together point toward potential revisions of how cervicovaginal dysbiosis is understood in HPV natural history.

The cervicovaginal mycobiome structure diverges significantly by HPV status, with an elevated *Ascomycota*/*Basidiomycota* ratio and, at finer resolution, a higher *Pichiomycetes*/*Tremellomycetes* ratio in HPV-positive women (*p* ≤ 0.001). The fungal restructuring co-occurs with HPV status but not with cytological category, a temporal ordering compatible with mycobiome change being an early feature of the HPV natural history rather than as a secondary consequence of cervical lesion development [35]. *Candida* colonization is associated with HPV, and in particular with the high-risk α-9 clade, at magnitudes not previously reported at this resolution. In cytologically normal women, *Candida*-positive status raised the odds of any HPV detection by a factor of 3.6 (*p* ≤ 0.01) and of α-9 HPV by a factor of 5.3 (*p* ≤ 0.01). This is compatible with a role for *Candida* being a candidate early correlate of HPV positivity rather than as a sequela of cervical lesions, although the cross-sectional design does not allow this distinction to be tested directly. Linking these observations to the bacterial side of the community, the community state type is linked to mycobiome composition through functionally characterizable metabolic repertoires. *L. iners*-dominated CST III carries the *Lactobacillus* label but less than two-times reduced prevalence of the H_2_O_2_-generating pyruvate oxidase gene K00158 (12.2%), an order of magnitude below *L. crispatus*-dominated CST I (34.0%) and a 3-fold increase in *C. albicans* prevalence. At species level, the *Gardnerella vaginalis*-dominated CST carries the strongest associations in the entire dataset with both *C. albicans* (q < 0.05) and *C. dubliniensis* (q < 0.05) prevalence. Neither the genus-level *Candida* aggregate nor *C. orthopsilosis* reaches significance in that community, so the *G. vaginalis* association is species-specific rather than a broader *Candida* phenomenon.

The taxonomic and functional readouts point in the same direction, and the combined picture changes how *L. iners* should be read. The protective phenotype of the *Lactobacillus*-dominated cervicovaginal niche rests on three processes—acidification via lactate, oxidative inhibition via H_2_O_2_, and competitive exclusion at the epithelial surface—and our functional data show that *L. iners* communities decouple them. *L. iners*-dominated communities retain acidification capacity through L-lactate (78.6% of all lactate metabolism genes fell for K00016) but show depleted gene capacity for H_2_O_2_ generation (K00158 collapsed to 12.2%) and for D-lactate biosynthesis (K03778 at 9.2%, against 17.0% in CST I). Hydrogen peroxide is a direct antifungal effector at concentrations achievable in the *L. crispatus*-dominated niche [21,36], and D-lactate, beyond its role in pH homeostasis, inhibits matrix metalloproteinase activity involved in both fungal invasion and epithelial remodeling of relevance to HPV [19,20]. A community whose gene content predicts predominantly L-lactate production, with limited oxidative capacity, is the bacterial context in which we observe a permissive fungal environment empirically: fungal prevalence approaching dysbiotic CST IV-B levels despite nominal *Lactobacillus* dominance. This interpretation aligns with the body of evidence that *L. iners* behaves as an atypical *Lactobacillus*. The species has a markedly reduced genome with exclusive L-lactate production and no D-lactate [16] and features documented across multiple strains [17,37,38]. Our observation that *L. iners* communities are enriched in the KEGG bacterial secretion system pathway is consistent with this profile and suggests that CST III should not be treated as an intermediate between CST I and CST IV. It occupies a distinct, fungus-permissive state, a reading supported by recent integrative metagenomic–metatranscriptomic work on the vaginal niche [23].

A biological explanation for the strong *Candida*-α-9 HPV association in cytologically normal women can be offered from what is known about *C. albicans* interaction with the cervicovaginal epithelium. *C. albicans* produces candidalysin, a 31-amino-acid peptide cleaved from the *ECE1* gene product, which permeabilizes epithelial membranes and activates pro-inflammatory signaling [24,25,26,39]. A mucosal surface repeatedly exposed to *C. albicans* in this active form may, therefore, present a lower effective barrier to HPV acquisition and persistence, particularly for α-9 high-risk types, whose transmission requires microtrauma and access to basal epithelial cells [39]. Our cross-stratum findings are compatible with this reading: the *Candida*-HPV association is strongest precisely in NILM women, where overt cytological damage has not yet occurred, and strongest specifically for α-9 HPV rather than for HPV of any type. A compatible pattern emerges from the large Chinese cohort of Chen and colleagues [28], who reported a dual effect of *Candida* on HPV: protective against HPV acquisition cross-sectionally (OR = 0.92) [40] but promoting HPV persistence longitudinally among already-infected women (HR = 1.77). The exploratory *ECE1* detection in our data (*n* = 8 *ECE1*-positive; *n* = 17 *ECE1* plus *SAP* family genes positive) shows that candidalysin-encoding sequences are recoverable from unselected shotgun metagenomic data, although the absolute yield is small and supports only proof of concept for future targeted profiling. A second immunological dimension complements this barrier-based interpretation. Candidalysin activates the epithelial NLRP3 inflammasome and IL-1α/IL-1β release [41], generating a sustained pro-inflammatory milieu that has been linked to attenuated IFN-λ antiviral signaling and enhanced basal epithelial proliferation, conditions compatible with HPV establishment and persistence [42]. Direct measurement of mucosal cytokines and IFN-λ activity in *Candida*-positive women, beyond the scope of the present design, will be required to test this mechanism.

Placing these findings in an international context, the cervicovaginal mycobiome has now been profiled by amplicon sequencing, shotgun metagenomics, and metaproteomics across several geographic populations. Table 2 (Section 2.3) summarizes six published studies alongside the present cohort, and two patterns stand out when the dominant genera are grouped by their higher-level taxonomy. The first is the stability of *Ascomycota*, and specifically the genus *Candida*, as a core component of the cervicovaginal mycobiome across all populations examined: *Candida* is a dominant or subdominant genus in every study in Table 2, from the asymptomatic Estonian cohort [14] through to the South African metaproteomic survey, in which *Candida* reached 53.2% of relative protein abundance. Within this *Ascomycota* core, our covariate-adjusted MaAsLin3 analysis resolves species-level structure: it carries distinct association profiles that earlier genus-level ITS reporting would have collapsed and indicates that the bacterial–fungal dependencies within the *Candida* genus are species-specific rather than uniform. The Caribbean Hispanic cohort [30] is dominated in its basidiomycete fraction by *Sporidiobolaceae* (class *Microbotryomycetes*) and *Malassezia* (class *Malasseziomycetes*); the South African cohort shows *Malassezia* enrichment in bacterial vaginosis [15]; data demonstrates that our Kazakhstani cohort, by contrast, carries *Cryptococcus* (class *Tremellomycetes*) as its principal basidiomycete component and, at the class level, shows the *Pichiomycetes*/*Tremellomycetes* ratio as its most sensitive HPV-associated fungal marker. Several explanations for this geographic variation are plausible and non-exclusive. Reference database coverage and taxonomic resolution differ across studies; shotgun metagenomics using expanded fungal catalogs outperforms ITS-based surveys at the species level into a single *Candida* signal. Environmental exposure also differs substantially between Caribbean coastal, South African urban/periurban, and Central Asian continental populations, and dietary fermented products (kumys, shubat) with documented yeast content are distinctive to the Central Asian setting. Host genetic variants affecting fungal recognition, such as MBL2, FCN2, and CARD9, may favor colonization by different fungal taxa across ancestries, and systematic inter-ancestry comparison has not yet been undertaken for the cervicovaginal niche. The value of positioning the Kazakhstani data in this international context is not primarily comparative but integrative: the cross-kingdom *Candida*-HPV association we report, anchored for α-9 HPV and OR = 5.3 in NILM, rests on a core *Ascomycota* signal reproducible across continents [15,27,28,43], while the regionally specific *Tremellomycetes* prominence flags where the cervicovaginal mycobiome literature has yet to reach.

The strengths of this study are four-fold: it provides the first shotgun metagenomic characterization of cross-kingdom cervicovaginal dysbiosis in a Central Asian cohort and addresses a substantial geographic gap in the literature; the cohort size (*n* = 311) is substantial for a cervicovaginal mycobiome study; the combined PCR HPV-mNGS detection strategy improved sensitivity by 25% over PCR alone and provided well-powered HPV stratification; single-assay profiling of bacterial, fungal, and viral compartments from the same shotgun dataset removes cross-platform biases that complicate cross-kingdom comparisons in studies combining 16S, ITS, and separate virome protocols; and the integration of taxonomic classification with KEGG-based functional annotation, including gene-level analysis of K00016, K00158, and K03778, provides biological interpretation beyond compositional description. Several limitations must be acknowledged. The cross-sectional design cannot resolve causal direction. Whether *Candida* colonization facilitates HPV acquisition or whether HPV infection produces a mucosal environment that favors fungal expansion remains undetermined, and prospective longitudinal cohorts will be needed. The cohort lacks HSIL and invasive cancer categories, so generalizability to later stages of cervical neoplastic progression cannot be assumed and will require confirmation in cohorts enriched for advanced cytology. Without culture-based validation, our quantitative fungal estimates depend on the completeness of reference databases, and rare or low-abundance taxa outside the core *Candida* and *Saccharomyces* genera may be affected by database gaps or classification artefacts. A related limitation concerns the manual curation step itself (Section 2.6). By restricting downstream analyses to 26 of 48 observed genera on the basis of documented human host association, we prioritized specificity over breadth and accepted the risk that poorly characterized but genuine cervicovaginal residents may have been excluded, including anthropophilic strains nested within otherwise environmental genera and under-investigated *Basidiomycota* whose mucosal niche has not yet been described. This risk is amplified by the geographic skew of the existing cervicovaginal mycobiome literature (Table 2), in which Central Asian populations are absent: a documented human association precedent may simply not yet exist for taxa that are genuine residents in our cohort. The full genus-level retention table is provided in Supplementary Table S1 to enable re-analysis under alternative criteria, and ITS-targeted sequencing or culture-based isolation in future cohorts will be required to resolve whether any excluded genera represent true under-characterized members of the niche. The absence of metabolomic and transcriptomic profiling means that the proposed processes (L-lactate, D-lactate, H_2_O_2_, candidalysin) are inferred from gene presence rather than directly measured, and functional potential does not always translate to functional activity. The *ECE1* (candidalysin) detection analysis is exploratory and based on a small number of positive samples (*n* = 8), which is insufficient for robust statistical inference; quantitative evaluation of candidalysin’s contribution to cervicovaginal dysbiosis will require targeted enrichment or transcriptomic protocols designed for this gene family in future cohorts. The viromic compartment is limited to quantitative assessment of HPV; systematic profiling of the broader cervicovaginal virome, including anelloviruses, herpesviruses, and phage communities, remains a task for future work. The cohort represents a single geographic region and predominantly one ethnic group, and replication in independent Central Asian and inter-ethnic cohorts will be necessary. Our sample is restricted to reproductive-age women (20–46 years), and peri- and post-menopausal cohorts, with their distinct estrogen-dependent cervicovaginal microbiome dynamics, are not represented. The cohort was recruited from a single clinical site in Astana and reflects the predominant ethnic composition of that catchment area. Extension to other Central Asian populations (Uzbek, Kyrgyz, Tajik, Turkmen) and to ethnically heterogeneous urban cohorts will be required to determine whether the *Candida*-HPV-α9 association we report generalizes across the region. A specific source of residual confounding warrants emphasis. Antimicrobial exposure history was captured only for the four weeks preceding sampling, whereas both systemic antibiotics and antifungals, particularly azoles, are documented to perturb the mycobiome on timescales of months to years, through direct selection on fungal taxa and through secondary effects mediated by depletion of bacterial commensals. Exposures outside this four-week window, therefore, cannot be excluded as contributors to the dysbiotic CST and *Candida*-positive states observed at sampling, and disentangling treatment-induced from spontaneous dysbiosis will require future cohorts with structured lifetime antimicrobial exposure histories or, ideally, electronic health record linkage. Adjustment for lifestyle, sexual behavior, and hormonal status was constrained by available metadata, and residual confounding cannot be fully excluded.

## 4. Materials and Methods

### 4.1. Study Design, Recruitment, and Ethics

This cross-sectional observational study was conducted between November 2024 and March 2025 at the outpatient gynecological clinic of University Medical Center (UMC), Astana, Kazakhstan. All women attending routine cervical screening during the study period were first assessed for eligibility by the attending clinician. Inclusion criteria were as follows: (i) age 18–60 years; (ii) routine cervical screening visit with no acute genitourinary symptoms at the time of sampling; (iii) no antibiotic or antifungal therapy in the four weeks preceding sampling; (iv) no vaginal douching or use of intravaginal products in the 48 h preceding sampling; and (v) written informed consent. Pregnant women and women who had undergone hysterectomy were excluded.

Of 753 women assessed for eligibility, 396 provided informed consent (participation rate 52.6%). After application of full inclusion criteria and quality control of biospecimens, 318 women entered the downstream analyses. Seven samples were additionally excluded from the primary cytology-stratified analyses because of unclassifiable cytological results, yielding a final analytical sample of *n* = 311 stratified across four Bethesda categories: NILM (*n* = 145), ASC-US (*n* = 75), LSIL (*n* = 81), and ASC-H (*n* = 10).

The study protocol was approved by the local ethics committee (2024/02-013 from 10 May 2024). All procedures were performed in accordance with the Declaration of Helsinki.

### 4.2. Sample Collection and DNA Extraction

Cervicovaginal swabs were collected during colposcopy using sterile urogenital probes (Jiangsu SuyunMedical Materials Co., Ltd., Lianyungang, China), transferred into 1.5 mL microtubes, and stored at −80 °C until processing. Total DNA was extracted using the ZymoBIOMICS DNA Miniprep Kit (Cat# D4300, Zymo Research, Irvine, CA, USA) following the manufacturer’s protocol. DNA quality and integrity were assessed using a combination of complementary methods: NanoDrop 2000 spectrophotometry (Thermo Fisher Scientific, Waltham, MA, USA) for purity (A260/A280 and A260/A230 ratios), Qubit 3.0 fluorometric quantification for concentration, and agarose gel electrophoresis for integrity. Extraction-negative controls were included in every batch and sequenced alongside clinical samples to enable contamination assessment.

### 4.3. HPV Detection and α-9 Genotyping

HPV status was determined via a combined approach using PCR-based genotyping and shotgun metagenomic sequencing (mNGS). A sample was classified as HPV-positive if at least one of the two methods detected HPV sequences. PCR genotyping was performed using (RealBest DNA HPV High Carcinogenic Risk Genotype kit, Cat# C8899, Vector-Best, Novosibirsk, Russia) targeting 16, 18, 31, 33, 35, 39, 45, 51, 52, 56, 58, and 59. From mNGS data, HPV detection was based on taxonomic read assignment to the genus *Alphapapillomavirus* and species-level resolution where read depth permitted. HR-HPV was determined based on α-9 HPV-positivity, defined as detection of any member of the *Alphapapillomavirus* 9 phylogenetic group (HPV-16, -31, -33, -35, -52, -58), a clinically relevant high-risk cluster.

The PCR-based approach identified HPV in 111 samples. The mNGS approach identified HPV in 120 samples. Agreement between approaches was 78.46%. The combined PCR-mNGS approach expanded the HPV-positive cohort from 111 samples (PCR alone) to 149 samples and provided the basis for all subsequent HPV-stratified analyses. Similarly, the PCR-based approach identified high-risk α-9 HPV in 81 samples, and the mNGS approach identified it in 71 samples. Agreement between approaches was 86.5%. The combined PCR-mNGS approach yielded 97 α-9 HPV-positive samples. The combined PCR-mNGS approach yielded 97 α-9 HPV-positive samples, which were used for subsequent analyses. Finally, PCR detected 34 samples with HPV16, while mNGS detected 43. The agreement reached 90.7%, and, in total, 53 samples were marked as HPV16-positive.

### 4.4. Shotgun Metagenomic Sequencing

Library preparation and shotgun metagenomic sequencing were performed by Novogene Co., Ltd. (Beijing, China) on the Illumina NovaSeq 6000 platform in paired-end mode (2 × 150 bp), with a minimum sequencing depth of 6 Gb per sample to ensure sufficient coverage of low-abundance taxa, including the fungal compartment. Sequencing yielded a median of 45.6 million paired-end reads per sample (range 39.6–71.4 million).

### 4.5. Bioinformatic Pipeline

The raw sequencing reads were processed and analyzed on a high-performance computing system. Reads were quality-controlled and adapter-trimmed with fastp (v0.23, default parameters with additional poly-G trimming for NovaSeq data). Host (human) read depletion was performed using Bowtie2 v2.5.4 by competitive alignment against the GRCh38 reference genome, retaining only reads that failed to align.

Taxonomic classification of the remaining microbial reads was performed using Kraken2 v2.1.3 against the PlusPFP database. We retained the default confidence threshold of 0.0 as the primary classification setting and instead controlled false-positive risk through downstream filtering, for two reasons. First, increasing the Kraken2 confidence threshold preferentially discards reads from low-abundance taxa and from genera that are under-represented or unevenly covered in the reference database—a behavior that is acceptable for high-biomass samples but disproportionately erodes signal in low-biomass compartments such as the cervicovaginal mycobiome [44]. Second, for fungi specifically, available reference genomes remain sparse relative to bacterial references, so higher thresholds preferentially penalize true but lineage-poor fungal hits. To compensate for the resulting permissiveness, false-positive control was implemented downstream in two layers: (i) genus-level manual curation restricting analyses to taxa with documented human host association (Section 2.6) and (ii) restriction of biological inference to genera/species consistently detected across the cohort. Fungal-classified reads, after host and bacterial read removal, were sparse, with a median of 132.0 reads per sample (IQR: 104.0–192.5), consistent with the low fungal biomass characteristic of the cervicovaginal niche in the absence of targeted fungal enrichment.

Functional profiling was performed with eggNOG-mapper v2 against the eggNOG database (v5.0.2), with subsequent regrouping to KEGG Orthology (KO) identifiers and pathway-level aggregation using the KEGG BRITE hierarchy.

### 4.6. Fungal Filtering: Human-Associated Taxa

To minimize the contribution of environmental-, plant- and soil-derived organisms, for which cervicovaginal residency is largely unsupported, the genus-level fungal taxonomy table from Kraken2 was manually curated prior to downstream analysis. Each observed genus was classified, on the basis of its primary ecological niche and documented host range, as either human-associated (retained) or environmental (excluded). Human-associated genera comprised members with established human commensal, mucosal, cutaneous, or opportunistic-pathogen status; environmental genera comprised obligate plant pathogens, soil saprotrophs, thermophilic compost-associated fungi, mycorrhizal symbionts, entomopathogens, and herbivore-dung-associated taxa. For two genera (*Vanrija*, *Botrytis*), retention was justified on the basis of recent studies documenting their occasional role in human colonization or infection (Supplementary Table S1).

Of 48 fungal genera observed across the cohort, 26 were retained for downstream analyses and 22 were excluded. A complete list of observed fungal genera with their inclusion status and rationale is provided in Supplementary Table S1.

### 4.7. Community State Type (CST) Classification

Bacterial CSTs were determined based on the most abundant species in the sample. The following dominant *Lactobacillus* species were considered: CST I: *L. crispatus*; CST II: *L. gasseri*; CST III: *L. iners*; CST V: *L. jensenii*. Samples not dominated by *Lactobacillus*—mostly *Gardnerella*-dominated communities—were designated as CST IV (subdivided into *G. vaginalis*-dominated and other-dominated types).

### 4.8. Statistical Analysis

All statistical analyses were performed in R version 4.5.3 and Python 3.12. Alpha diversity of the fungal community was summarized by observed species richness and Pielou’s evenness. Beta diversity was assessed on Bray–Curtis distances of Hellinger-transformed fungal abundances. Group separation was tested with PERMANOVA (999 permutations) and dispersion homogeneity with PERMDISP (999 permutations), the latter to distinguish true centroid shifts from dispersion artefacts. Ordination was visualized with principal coordinates analysis (PCoA).

Phylum- and class-level ratios (*Ascomycota/Basidiomycota*; *Pichiomycetes*/*Tremellomycetes*) were computed per sample after adding a 1 pseudocount to avoid division by zero and compared between HPV groups via Mann–Whitney U test.

KEGG pathway and gene analyses.

Pathway-level relative abundances (ko00620 pyruvate metabolism; ko03070 bacterial secretion system) were compared across abundant bacterial CSTs (CST III and CST IV (*G. vaginalis* dominated) using the Mann–Whitney U test; *L. crispatus* served as the reference group in pairwise contrasts. KEGG Orthology gene relative presence was summarized as the median gene ratio in each CST carrying the gene for three functional markers of bacterial protective capacity: K00016 (L-lactate dehydrogenase), K00158 (pyruvate oxidase, H_2_O_2_-generating), and K03778 (D-lactate dehydrogenase). The presence of the *Candida albicans* candidalysin-encoding gene *ECE1* and associated genes (*TUP1*, *EFG1*, *KEX1*, *KEX2*, *UME6*, *BCR1*, *BRG1*, *ALS3*, *HWP1*, *SAP4*, *SAP5*, *SAP6*) was estimated from eggNOG gene-level annotation.

Statistical analyses followed a two-layer strategy. Differential prevalence of a set of pre-selected variables was first assessed using Fisher’s exact test without correction for multiple comparisons and is reported as unadjusted odds ratios with 95% confidence intervals. Second, covariates’ effects on differential prevalence were examined using MaAsLin3 regression analysis (described below), which provided covariate-adjusted estimates with Benjamini–Hochberg FDR correction. Results from both layers are reported in parallel throughout the manuscript so that unadjusted and adjusted effects can be distinguished.

Associations between prevalence of fungal taxa and covariates were tested using MaAsLin3 v1.2.0 using only prevalence model, adjusting for age, BMI, marital status, number of sexual partners, parity, cervical erosion, menstrual cycle phase, and cell status. Multiple testing was controlled with the Benjamini–Hochberg procedure; taxa with FDR-BH < 0.05 were retained as candidate associations.

Bacterial community composition and demographics were compared across HPV status using Mann–Whitney U test, Fisher’s exact test, or chi-squared tests as appropriate. The significance threshold for all analyses was *p* < 0.05 except where multiple testing correction was applied (FDR < 0.05 for MaAsLin3).

## 5. Conclusions

In this shotgun metagenomic study of 311 Kazakhstani women, the first cervicovaginal mycobiome characterization from Central Asia, we report four linked findings. HPV status is associated with a restructuring of the fungal compartment, with the *Pichiomycetes*/*Tremellomycetes* class-level ratio as the most sensitive marker (*p* ≤ 0.001). *Candida* colonization is associated with HPV and α-9 HR-HPV detection (OR = 3.6 and OR = 5.3) in women with normal cytology, and the covariate-adjusted analysis confirms this pattern. Species-level resolution enabled by mNGS reveals heterogeneity of fungal–bacterial interactions, distinctions that genus-level reporting would collapse. *Gardnerella vaginalis*-dominated CST shows elevated prevalence of both *C. albicans* and *C. dubliniensis*. At the same time, *L. iners*-dominated CST III shows a 3-fold higher *C. albicans* prevalence. Finally, our functional data show that the *L. iners*-dominated community carries half the H_2_O_2_-generating pyruvate oxidase gene capacity of the *L. crispatus*-dominated CST I, which, along with the elevated prevalence of *C. albicans*, supports a reading of *L. iners* as a functionally distinct, fungus-permissive *Lactobacillus* rather than a weaker *L. crispatus* sibling. Placed alongside the six other cervicovaginal mycobiome studies summarized in Table 2, our data confirm *Candida* (*Ascomycota*) as a universal component of the cervicovaginal mycobiome and provide the first detailed characterization of this niche in Kazakhstani women.

## Figures and Tables

**Table 1:** Demographic and clinical characteristics by cytological severity group.

Variable	Level	HPV-Negative (*n* = 162)	HPV-Positive (*n* = 149)	*p*-Value
α-9 HR-HPV Status, *n* (%)		-	97 (65.1%)	
HPV16 Status, *n* (%)			53 (35.6%)	
Age yrs., Md (IQR)		35.32 (IQR: 31.69; 39.96)	34.17 (IQR: 28.47; 38.01)	*p* ≤ 0.05 ^a^
BMI, Md (IQR)		23.06 (IQR: 20.57; 25.37)	22.6 (IQR: 20.31; 25.8)	*p* = 0.52 ^a^
Kazakh, *n* (%)		145 (91.2%)	132 (90.4%)	OR = 0.9 [0.4–2.0], *p* = 0.86 ^b^
Menarche, Md (IQR)		13.0 (IQR: 13.0; 14.0)	13.0 (IQR: 13.0; 14.0)	*p* = 0.46 ^a^
Parity, Md (IQR)		2.0 (IQR: 1.0; 3.0)	1.0 (IQR: 0.0; 2.0)	*p* ≤ 0.001 ^a^
Married, *n* (%)		121 (74.7%)	81 (55.5%)	OR = 0.4 [0.3–0.7], *p* ≤ 0.001 ^b^
Partners, Md (IQR)		1.0 (IQR: 1.0; 1.0)	1.0 (IQR: 1.0; 2.0)	*p* = 0.24 ^a^
Erosion, *n* (%)		84 (51.9%)	66 (44.3%)	OR = 0.7 [0.5–1.2], *p* = 0.21 ^b^
Vaginosis, *n* (%) **		57 (35.2%)	61 (40.9%)	OR = 1.3 [0.8–2.1], *p* = 0.35 ^b^
Bacterial CST, *n* (%)				
	CST I (*L. crispatus*)	38 (23.5%)	31 (20.8%)	*p* = 0.79 ^c^
	CST II (*L. gasseri*)	17 (10.5%)	11 (7.4%)	
	CST III (*L. iners*)	44 (27.2%)	42 (28.2%)	
	CST IV (*G. vaginalis*)	25 (15.4%)	30 (20.1%)	
	CST IV (Other *G*. sps.)	32 (19.8%)	31 (20.8%)	
	CST V (*L. jensenii*)	6 (3.7%)	4 (2.7%)	
Cervical Disease Status, *n* (%)				
	NILM	103 (63.6%)	42 (28.2%)	OR = 4.4 [2.7–7.4], *p* ≤ 0.0001 ^b^
	non-NILM	59 (36.4%)	107 (71.8%)	
	ASCUS	39 (24.1%)	36 (24.2%)	*p* ≤ 0.0001 ^c^
	LSIL	20 (12.3%)	61 (40.9%)	
	ASCH	0 (0%)	10 (6.7%)	
*Candida* Status, *n* (%)		74 (45.7%)	95 (63.8%)	OR = 2.1 [1.3–3.4], *p* ≤ 0.001 ^b^
*Candida albicans* Status, *n* (%)		48 (29.6%)	61 (40.9%)	OR = 1.6 [1.0–2.7], *p* ≤ 0.05 ^b^
*Candida dubliniensis*, *n* (%)		22 (13.6%)	40 (26.8%)	OR = 2.3 [1.3–4.4], *p* ≤ 0.01 ^b^
*Saccharomyces*, Status, *n* (%)		61 (37.7%)	79 (53.0%)	OR = 1.9 [1.2–3.0], *p* ≤ 0.01 ^b^
*Saccharomyces cerevisiae*, Status, *n* (%)		12 (7.4%)	27 (18.1%)	OR = 2.8 [1.3–6.2], *p* ≤ 0.01 ^b^

Note: ** non-*Lactobacillus* dominated, ^a^—Mann–Whitney U test; ^b^—Fisher’s exact test; ^c^—Chi-squared. All percentages are computed column-wise as proportions within HPV-negative or HPV-positive groups. All comparisons in this table are descriptive. The *p*-values are calculated without adjustment for multiple testing, and no additional covariates are accounted for except for the one described.

**Table 2:** Cross-population comparison of published cervicovaginal mycobiome studies.

#	Population	*n*	Method	Dominant Fungal Genera	Phylum/Major Taxonomic Group	Key Fungal/HPV Finding	Reference
1	Estonian (asymptomatic, reproductive-age)	494 total; 216 with ITS	16S + ITS-1 pyrosequencing (454)	*Candida*, *Saccharomyces*	*Ascomycota* (*Saccharomycetes*)	First NGS characterization of vaginal mycobiome; 196 fungal OTUs, 16 *Candida* OTUs	Drell et al. 2013 [14]
2	Puerto Rican Hispanic (high-risk clinics)	62 total; 55 (cervix)	16S V4 + ITS-2 (Illumina)	*Sporidiobolaceae*, *Malassezia*, *Candida*, *Saccharomyces*	*Basidiomycota* (*Sporidiobolaceae*: *Microbotryomycetes*; *Malassezia*: *Malasseziomycetes*) + Ascomycota (*Candida*, *Saccharomyces*: *Saccharomycetes*)	Fungal Shannon diversity ↑ in HR-HPV+ cervix (*p* = 0.05); Sporidiobolaceae dominant in ASC-US and low-risk HPV; Malassezia biomarker for HR-HPV	Godoy-Vitorino et al. 2018 [31]
3	Costa Rican (CVT trial; incident HR-HPV, 18–25 y)	273 (266 longitudinal)	16S V4 + ITS-1 (Illumina MiSeq)	*Ceriporia lacerata*, *Aspergillus conicus*, *Aspergillus caespitosus*	*Basidiomycota* (*Ceriporia lacerata*: *Irpicaceae*) + *Ascomycota* (*Aspergillus conicus*; *Aspergillus caespitosus*: *Eurotiomycetes*)	Fungal diversity protective against CIN2+ progression (OR = 0.90, 95% CI 0.82–1.00)	Usyk et al. 2020 [27]
4	Thai (HPV16 + HR-HPV with CIN1/2/3)	47	bacterial + fungal + viral community sequencing	*Candida albicans*	*Ascomycota* (*Saccharomycetes*)	Few fungi detected at species level; HPV reduces viral diversity	Sasivimolrattana et al. 2022 [32]
5	Korean (HPV16/18 vs. Other HR-HPV; normal, LSIL, HSIL, ICC)	68	Shotgun metagenomics (MGI DNBSEQ-G400RS) + 137 MAGs	Fungal genera not explicitly detailed; multi-kingdom network analysis	Not specified at phylum level in original paper	HPV16/18 exhibits stronger bacterial–fungal inter-kingdom correlations than other HR-HPVs in groups 1 and 3	Jung et al. 2025 [33]
6	Kazakhstani (Central Asian, cervical screening)	311	Shotgun metagenomics (Illumina NovaSeq 6000)	*Candida*, *Cryptococcus*	*Ascomycota* (*Candida*: *Saccharomycetes*) + *Basidiomycota* (*Cryptococcus*: *Tremellomycetes*)	*Candida* prevalence demonstrates an association with α-9 HPV occurrence OR = 5.3 [1.6–69.5] in NILM (*p* ≤ 0.01); Class-level ratio of *Pichiomycetes* to *Tremellomycetes* appears as the most sensitive HPV marker (*p* ≤ 0.001); Co-occurrence of *L. iners* with *Candida* suggests that *L. iners* is functionally permissive	Present study

## Data Availability

The data from this study have been deposited in the NCBI BioProject database under the accession number PRJNA1337009. All datasets are publicly accessible through this repository.

## Supplementary Materials

The following supporting information can be downloaded at: https://www.mdpi.com/article/10.3390/ijms27115052/s1.
