# Supplementary material for: Cervicovaginal Mycobiome Restructuring by HPV and Bacterial Community State Types in a Kazakhstani Shotgun Metagenomic Cohort: Lactobacillus iners as a Candida-Permissive Niche Associated with α-9 HPV in Cytologically Normal Women
Source: Int J Mol Sci. 2026 Jun 3;27(11):5052. doi: 10.3390/ijms27115052 (PMC13256560; doi:10.3390/ijms27115052)
Supplement: Supplementary file 1 [file ijms-27-05052-s001.zip › ijms-4323061-supplementary.pdf]

Table S1. Fungal genera observed in the Kazakhstani cohort (n=311) and their inclusion status for downstream cervicovaginal mycobiome analyses, classified by primary ecological niche.

**All observed genera**	Included/excluded	
Candida	Included	
Saccharomyces	Included	
Cryptococcus	Included	
Malassezia	Included	
Lodderomyces	Included	
Schizosaccharomyces	Included	
Nakaseomyces	Included	
Coccidioides	Included	
Zygosaccharomyces	Included	
Torulaspora	Included	
Yarrowia	Included	
Naumovozyma	Included	
Ascochyta	Included	
Kluyveromyces	Included	
Vanrija	Included	https://doi.org/10.5145/ACM.2024.27.1.5
Botrytis	Included	https://doi.org/10.1186/s12879-019-4319-2
Fusarium	Included	
Brettanomyces	Included	
Cutaneotrichosporon	Included	
Rhizoctonia	Included	
Pichia	Included	
Debaryomyces	Included	
Scheffersomyces	Included	
Ogataea	Included	
Kazachstania	Included	
Lachancea	Included	
Kwoniella	excluded	primary vegetation, but phylogenetically close to Cryptococcus
Rhizophagus	excluded	
Neurospora	excluded	primarily soil, vegetation
Trichoderma	excluded	primarily soil
Colletotrichum	excluded	primary vegetation
Podospora	excluded	environmental
Sordaria	excluded	environmental
Marasmius	excluded	primary vegetation
Psilocybe	excluded	primary vegetation
Puccinia	excluded	primarily soil, vegetation
Mycosarcoma	excluded	primary vegetation
Sporisorium	excluded	primary vegetation
Metarhizium	excluded	
Akanthomyces	excluded	
Drechmeria	excluded	
Purpureocillium	excluded	
Pyricularia	excluded	
Thermothelomyces	excluded	
Thermothielavioides	excluded	
Remersonia	excluded	
Vairimorpha	excluded	parasitic unicellular fungi
Ustilaginoidea	excluded	
